# Opportunities for Persistent Luminescent Nanoparticles in Luminescence Imaging of Biological Systems and Photodynamic Therapy

**DOI:** 10.3390/nano10102015

**Published:** 2020-10-13

**Authors:** Douglas L. Fritzen, Luidgi Giordano, Lucas C. V. Rodrigues, Jorge H. S. K. Monteiro

**Affiliations:** 1 Department of Fundamental Chemistry, Institute of Chemistry, University of São Paulo, São Paulo-SP 05508-000, Brazil; douglas.fritzen@usp.br (D.L.F.); luidgi@iq.usp.br (L.G.); 2 Department of Chemistry, Humboldt State University, Arcata, CA 95521, USA

**Keywords:** persistent luminescence, luminescence imaging, theranostics, photodynamic therapy

## Abstract

The use of luminescence in biological systems allows us to diagnose diseases and understand cellular processes. Persistent luminescent materials have emerged as an attractive system for application in luminescence imaging of biological systems; the afterglow emission grants background-free luminescence imaging, there is no need for continuous excitation to avoid tissue and cell damage due to the continuous light exposure, and they also circumvent the depth penetration issue caused by excitation in the UV-Vis. This review aims to provide a background in luminescence imaging of biological systems, persistent luminescence, and synthetic methods for obtaining persistent luminescent materials, and discuss selected examples of recent literature on the applications of persistent luminescent materials in luminescence imaging of biological systems and photodynamic therapy. Finally, the challenges and future directions, pointing to the development of compounds capable of executing multiple functions and light in regions where tissues and cells have low absorption, will be discussed.

## 1. Introduction

**Figure 1:**
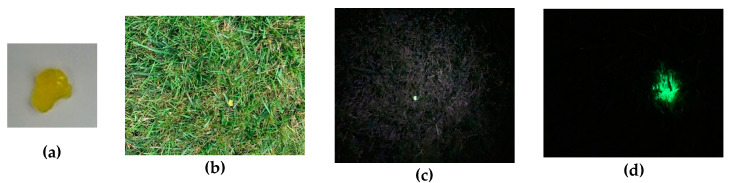
(**a**) Polymer under white light illumination. (**b**) Polymer dispersed in the grass under white light, analog to the cell staining technique. (**c**) Polymer dispersed in the grass under UV light, analog to the luminescence imaging technique. (**d**) Persistent luminescent material dispersed in the grass.

Observation of cells and the different cellular components is a fascinating field that allows one to diagnose diseases and unravel biological processes [1,2,3,4,5,6,7,8,9,10,11,12,13]. The simplest way to observe cellular components is using a simple optical microscope and color staining [14]. This technique, pioneered by C. Golgi and S. Ramon y Cajal, is based on color change caused by a specific dye [14]. Specific interactions between dye and tissue or dye and cellular components are capable of revealing details about tissue structures and cell components using inexpensive techniques. Color staining is a straightforward technique capable of providing intricate details about tissues and cells. However, it relies on specific interactions between dyes and tissues or cell components; the dye needs to be washed out to warranty specificity and usually requires high concentrations to allow acceptable color contrasts. As an analogy, imagine that the yellow polymer, shown in Figure 1a, is a dye used in cell staining, and the grass represents a cell. The cell staining technique consists of simply placing the polymer onto the grass. As shown in Figure 1b, it is hard to spot the polymer first and takes a well-trained set of eyes to do it. Now imagine that the polymer is luminescent under UV light exposure. The white light illumination is turned off, and the sample is excited using an adequate excitation wavelength (Figure 1c). The use of luminescence grants the reader a clear picture of the polymer’s location in the grass with no interference or low interference from the cell background. Thus, luminescence imaging of biological systems is based on exciting a volume of a sample containing a luminescent compound using an adequate excitation source and wavelength and collecting the light emitted. Luminescence imaging is a sensitive technique that allows diagnosing diseases [15,16,17], reconstructing 3-D structures of tissues or cellular components [18,19], sensing chemical species [1,2,3,12,20,21,22,23,24,25,26,27,28,29,30,31,32,33,34,35,36,37,38], and unraveling cellular processes [39,40,41]. One of the drawbacks of this technique is the strong background emission intensity, especially in the blue and green regions of the electromagnetic spectrum that are often higher than those of the luminescent compound.

Persistent luminescence (PeL) is a phenomenon where light is emitted for long periods, from minutes to hours, after the excitation, resulting in a glow-in-the-dark phenomenon [42,43,44,45,46,47,48,49,50,51,52,53,54]. If we come back to the analogy in Figure 1, now imagine that we use a material capable of luminescing without a continuous excitation. Using the same analogy that the grass represents a cell, we will achieve what is shown in Figure 1d. Because there is no continuous illumination, all the emission background is eliminated, and we can locate where the luminescent compound is located. Thus, the application of PeL materials eliminates the background emission and depth penetration problems, resulted from the excitation wavelengths in the UV-Vis, commonly used in luminescence imaging of biological systems [55,56,57,58,59,60,61,62,63,64,65,66,67,68,69,70,71,72].

Theranostics corresponds to systems capable of simultaneously treat (therapy) and diagnose (diagnostics) diseases. Recently, the research for non-invasive and tailored treatments have prompted research in treatments that involves the generation of heat (photothermal therapy, PTT), reactive oxygen species (photodynamic therapy, PDT) or gene therapy, to cite a few [43,73,74,75,76,77,78,79,80,81,82,83,84,85,86]. Photodynamic therapy (PDT) is a non-invasive therapy based on the generation of singlet oxygen (^1^O_2_) and/or reactive oxygen species (ROS). Cells and organisms are less likely to develop resistance to ^1^O_2_, making PDT attractive for treating cancer [79]. Organic dyes such as porphyrins, chlorins, phthalocyanines, and xanthenes are often used in PDT [87,88]. However, this class of compounds is prone to photobleaching, they have low light-dark cytotoxicity ratios, and they are also known to form aggregates that decrease the singlet oxygen generation efficiency as a function of the elapsed time, and thus decreases the efficiency of the treatment [89]. PeL materials are known to generate light. The possibility to use PeL materials in PDT is an exciting field that will render systems that do not to be excited throughout the treatment.

Due to the broad range of applications and promising use in luminescence imaging of biological systems, specific properties or specific applications of PeL materials have been reviewed over the past years. However, past reviews were focused solely on use and advances of PeL in biological systems [54,90], design and synthesis of PeL and their impact over the years [91]. Our work intends to go deeper into PeL nanomaterials applied for luminescence imaging in biological systems, their synthesis, and an extensive compilation of materials and methods for that specific application. Thus, this review aims to provide a background in luminescence imaging of biological systems, PeL, synthetic methods for obtaining PeL materials, and discuss selected examples of recent literature on the applications of PeL materials in luminescence imaging of biological systems and photodynamic therapy. The reader is referred to other reviews for detailed information about the persistent luminescence phenomenon and materials exhibiting this phenomenon [49,91,92,93].

## 2. Luminescence Imaging

**Figure 2:**
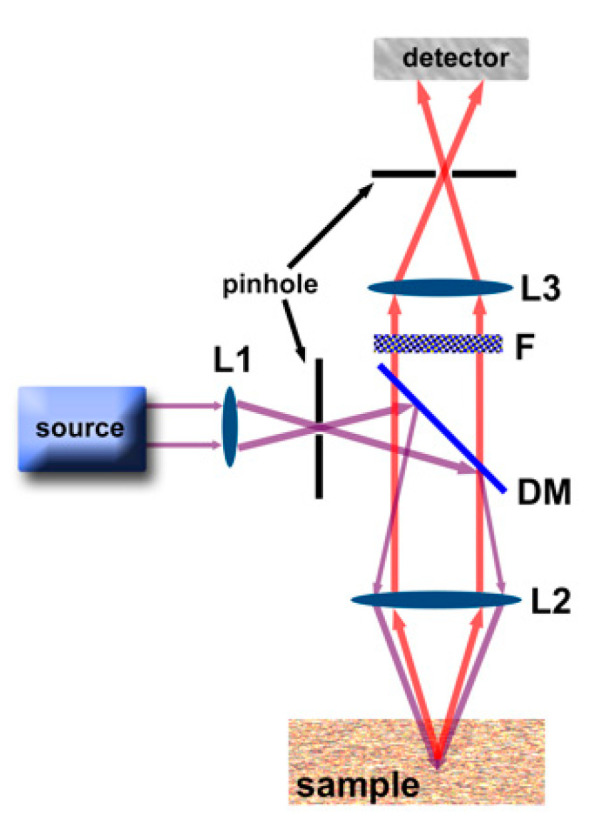
Confocal fluorescence microscope setup. L indicates lens, DM dichroic mirror, F filter, the purple and red lines indicate excitation and emission, respectively. Reproduced from [94] with permission from MDPI.

A simple scheme of a confocal fluorescence microscope is shown in Figure 2. The excitation light is first collimated by a set of lenses (L1), reflected by a dichroic mirror (DM), and excite the sample. The emission is then filtered by an adequate optical filter (F), collimated by a set of lenses (L3), and collected by the detector (a photomultiplier tube, or CCD) that transforms the photons in the electrical signal (Figure 2). Because the focus of this review is on persistent luminescence nanoparticles (PeL-NPs), we will not discuss the specifics of the function of the DM, lenses, and detectors. The reader is referred to the literature for more details about the fluorescence microscope components [14].

**Figure 3:**
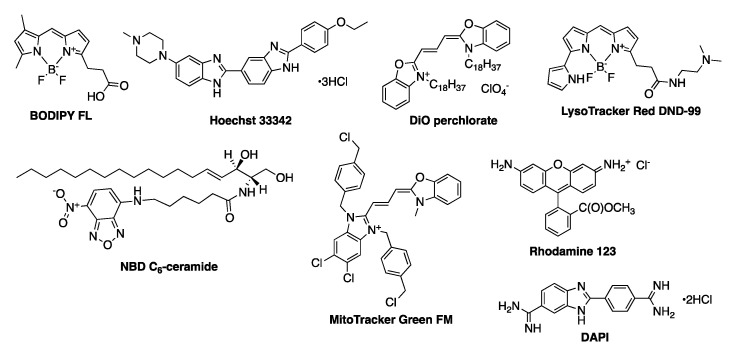
Structure of the most common dyes used for fluorescence cell staining.

**Figure 4:**
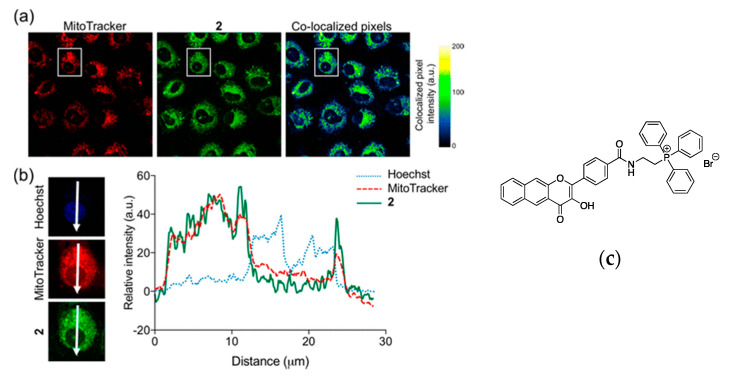
Cellular luminescence imaging of A549 cells. (**a**) From left to right, red emission of MitoTracker™ Red, green emission of the compound photoCORM-2, and overlay between the red and green channels. (**b**) The emission intensity of the blue, green, and red emissions as a function of the distance across the cell. (**c**) Structure of compound 2. The nucleus and mitochondria were stained with Hoechst 33342 and MitoTracker™ Red, respectively. [Hoechst 33342] = [MitoTracker™ Red] = 300 nM, [2] = 25–100 μM. Reproduced from [104] with permission from the American Chemical Society.

After being internalized by the cell, some luminescent labels accumulate in a specific organelle due to physical-chemical interactions [37,38,39,95,96,97,98,99,100,101,102,103,104,105,106,107]. One of the techniques used to determine in which organelle the luminescent label accumulates is the fluorescence co-localization experiment. In this experiment, the luminescent compound and a luminescent dye known to accumulate in a specific organelle are incubated in the cell together; the overlap of the emission intensity between the two compounds is then proportional to the accumulation of the luminescent label in the organelle. Ideally, the compound of interest and the dye used to tag a specific organelle have emission wavelengths in different regions of the electromagnetic spectrum that allow discriminating between the emission from each compound. A list with dyes for tagging specific organelles along with excitation and emission wavelengths, and their structures are shown in Table 1 and Figure 3, respectively. For example, fluorescence co-localization experiments were used to evaluate the mitochondria bioenergetics as a function of the CO delivery directly or indirectly to the mitochondria [104]. Using flavonol-based luminescent dye (Figure 4c) capable of releasing CO under illumination with visible light modified with a triphenylphosphonium (TPP) moiety that is known to cause accumulation in the mitochondria, the specific delivery of CO directly to the mitochondria was possible (Figure 4a,b). The study found that the specific and non-specific CO delivery has a similar effect on bioenergetics.

Another problem that arises, especially in the blue and green regions of the electromagnetic where the emission intensity from cells and tissues is high, is a strong background emission that will not allow the detection from the luminescent compound, especially when the compound has low emission. Some solutions to avoid the interference from the cell or tissue emission are red shifting the emission of the luminescent label to the red-NIR [13,109,110], use of two-photon absorption [111,112,113], upconversion emission [114,115], or use of emission lifetime mapping. In this Review, we will focus on the emission lifetime mapping measurement. The reader is redirected to the literature for a detailed description of luminescent labels with emission in the red-NIR, two-photon absorption, and upconversion materials [13,109,110,111,112,113,114,115,116].

**Figure 5:**
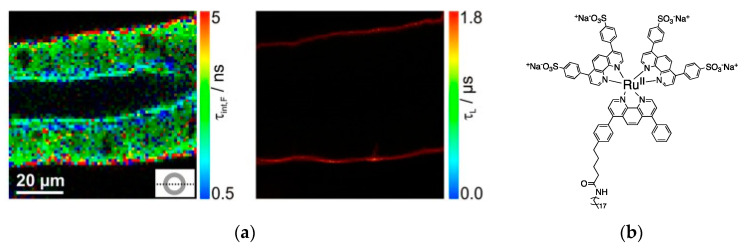
(**a**) Emission lifetime map of cockroach salivary ducts stained with a Ru^II^ complex using FLIM (left) or PLIM (right). (**b**) Structure of the Ru^II^ complex. Reproduced from [117].

The use of emission lifetime in cellular luminescence imaging is advantageous because it is reproducible. The emission lifetime is a non-extensive and specific property of each compound, allowing discrimination between the emission from the cell components and the luminescent label [117,118]. Cell components and organic dyes usually show emission lifetimes in the nanoseconds range, Table 2, which makes Fluorescence Lifetime Imaging Microscopy (FLIM) one of the most used techniques [119,120,121,122,123,124,125,126]. Although FLIM is a technique that allows us to discriminate between the emission lifetimes of the cell components and luminescent labels, there is not complete elimination of the cell emission from the image. Longer emission lifetimes, in the range micro-millisecond, can be achieved using transition metal complexes or lanthanide(III) compounds. These compounds show unique spin forbidden and/or Laporte forbidden, in the case of the Ln^III^ compounds, and are used in Phosphorescence Lifetime Imaging Microscopy (PLIM) [117,127,128,129,130,131,132]. Emission lifetimes higher than hundreds of nanoseconds allow complete elimination of the cell emission and yield a background-free image. For example, the FLIM emission lifetime map of cockroach salivary ducts does not allow to distinguish between cell components and the Ru^II^ complex (Figure 5a, left); the structure of the complex is shown in Figure 5b) [117]. Due to the emission lifetime in the microsecond range, the Ru^II^ complex the PLIM emission lifetime map can be obtained, providing a background-free image (Figure 5a, right) [117].

At this point, the reader has been presented with the potentialities and challenges in the luminescence imaging of biological systems. Although successful, luminescent organic dyes have several downfalls for using in luminescence imaging of biological systems such as short emission lifetime, small Stokes shift, and extensive photobleaching; all of those limitations leads to a not complete elimination of the emission background, interference of the excitation source in the imaging, and decrease of the emission intensity as a function of the time which does not allow for experiments with an extended period of time, respectively. Materials with long emission lifetimes such as lanthanide-doped nanoparticles, lanthanide complexes, and persistent luminescent materials are an alternative to the organic dyes for obtaining high-quality luminescence imaging. In this review, we will focus on persistent luminescent materials. The reader is directed to the literature for more details about lanthanide-doped nanoparticles and lanthanide complexes applications in luminescence imaging of biological systems [8,9,94].

## 3. Persistent Luminescence

Persistent luminescence (PeL) is a phenomenon where light is emitted for long periods of time, from minutes to hours, after the excitation resulting in a glow-in-the-dark phenomenon. Matsuzawa and co-workers were the first to report the SrAl_2_O_4_:Eu^2+^,Dy^3+^ green PeL emission that lasted >10 h, after being charged by UV light [141]. Research in PeL has flourished since then, and several examples based on doped/co-doped inorganic materials are found [49,91,92] with applications in emergency signage, road signalization, luminous paintings, temperature and pressure sensing [91,142], and cellular luminescence imaging [92], to cite a few.

### 3.1. PeL Mechanism

**Figure 6:**
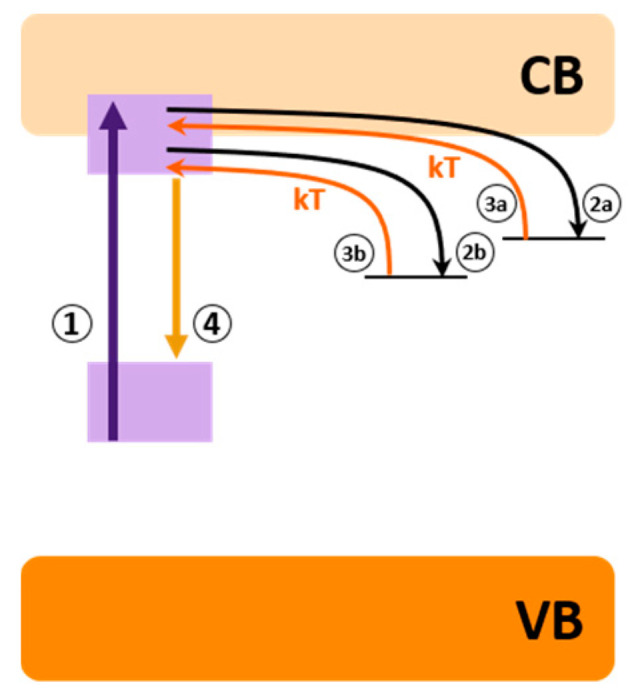
PeL simplified mechanism. VB is the valence band, CB conducting band, and kT is thermal energy.

Despite the long emission duration shared characteristic, phosphorescence and PeL are entirely different processes. While in phosphorescence, the long emission lifetime is caused by a spin-forbidden transition, in PeL the long emission time is caused by the storage of energy in traps [93] that are slowly promoted to the emitting levels. In these materials, the energy is stored by trapping charge carriers (electrons and/or holes), and it is slowly released with the aid of thermal energy. Thus, PeL is a particular case of thermostimulated luminescence [91] and is a defect dependent phenomenon. Although simple, the PeL full mechanism took several years to be figured out. The knowledge of trapping charge carriers (electrons and/or holes) in the defects for later thermal aid release dates back from 1939 when Johnson proposed the electron storage process to explain the ZnS PeL mechanism [143]. In 1945, Fonda observed that dopants and the crystalline phase influence the duration and intensity of PeL [144]. More detailed mechanisms, based on quantitative positioning of the energy levels and defects, appeared only in the 2000s with the works of Aitasalo and co-workers [145], Clabau and co-workers [146], and Dorenbos [147]. Nowadays, the PeL mechanisms for materials doped with Eu^2+^ or other similar emitters are very well established. This mechanism is summarized in four steps; the first step, centered in the activator, involves the excitation of the electrons (1), followed by trapping of the electrons into defects through the conducting band (CB) (2a) or directly via tunneling (2b). The trapped electron is then thermally promoted (kT) to the activator emitting levels via CB (3a), or via tunneling (3b), and finally decays radiatively, generating the PeL (4) (Figure 6).

The mechanism described above is just a general one, and variations of the excitation and trapping processes are known for different compositions. For example, in materials containing ions like Eu^2+^, Tb^3+^, and Ti^3+^, excitation to the *d* metal orbitals is enough to allow electron trapping [148,149], while in materials containing Eu^3+^ and Yb^3+^, only excitation to the charge transfer states allows the energy storage [150,151]. In materials containing ions like Cr^3+^, Mn^4+^, and Sm^3+^ [152,153,154], the primary excitation process that allows energy storage is the band gap excitation combined with energy transfer processes. The different excitation processes can be related to the emitting centers’ redox capacity since energy is stored by trapping electron or holes from the emitting center or the host. In the case of Eu^2+^-doped materials, it was already proven by X-ray absorption or EPR spectroscopy that in the charging process of persistent luminescence (process 1, Figure 6), Eu^2+^ is oxidized to Eu^3+^ [155,156].

**Figure 7:**
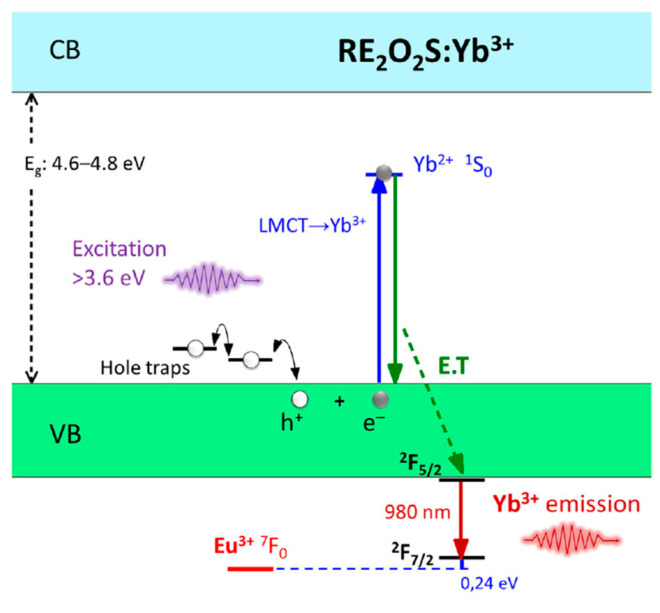
Yb^3+^-activated persistent luminescence mechanism in rare earth oxysulfides. Reproduced from [151] with permission from Elsevier.

The charge carriers trapping mechanism also changes for different compositions. Even if thermoluminescence experiments are good to quantify the defect concentration and to estimate the energy of the defects, there is no easy experiment to determine which charge carrier is participating in the process. Based on the idea of the energy level positions, the proposed mechanisms suggest that for most materials, like those doped with Ce^3+^, Eu^2+^, Tb^3+^, Cr^3+^, electron-trapping is the primary energy storage process. However, for materials dependent on ligand-to-metal charge transfer excitation as those doped with Eu^3+^ or Yb^3+^, hole trapping is the dominant energy storage process [150,151,157], (Figure 7). The hole trapping mechanism is similar to the electron mechanism where the storage happens under irradiation and the bleaching with thermal energy. However, the main differences are the defect type (must be negative in order to store holes), its position (close to the valence band) and finally, the excitation processes. The first excitation pathway is a band gap absorption followed by the trapping of both electrons and holes, which may occur with several emitting centers [91]. The second possible pathway is the charge-transfer excitation of a species followed by hole trapping leading to a reversible photoreduction of the species [151]. In this case, a metastable reduced form of the excited species is needed which is more probable when metals with low reduction potential are present, for example, Eu^3+^ → Eu^2+^ and Yb^3+^ → Yb^2+^ pairs.

The storage of both electron or holes occurs in point defects, mainly vacancies, self-interstitials, or substitutional ions (added as co-dopants). The vacancies and self-interstitials are formed either intrinsically or due to charge compensation via aliovalent doping. The formation of Schottky and Frenkel intrinsic defects is an endothermic process since it requires bond-breaking but has positive entropy variation due to gas formation combined to empty sites that increase the degrees of freedom of the material. Thus, high temperatures are needed to synthesize efficient PeL materials with high storage capacity in the intrinsic defects.

**Figure 8:**
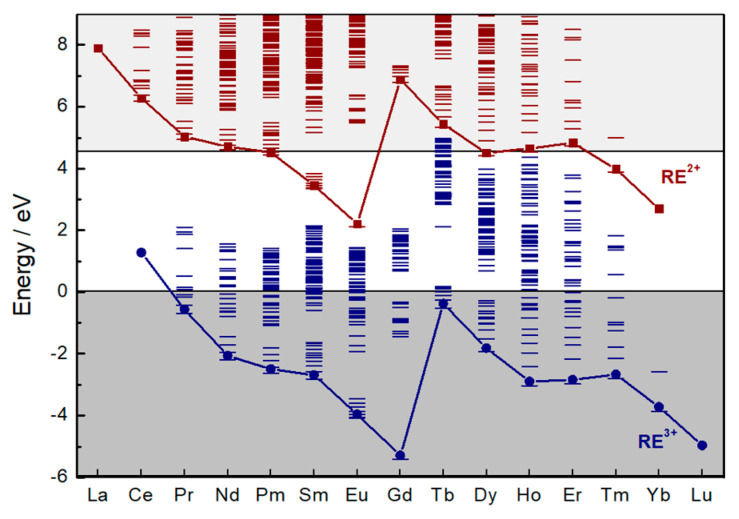
Host-referred 4*f*-electron binding energy curves and excited state energies of RE^2+^ and RE^3+^ ions in SrS. Reproduced from [156] with permission from The Royal Society of Chemistry.

Co-doping the system is another way to enhance energy storage capacity. When aliovalent co-doping takes place, charge compensations must take place to maintain the electric neutrality. For example, the PeL of SrS:Eu^2+^,RE^3+^ (RE = rare earth) materials is improved when different RE^3+^ ions replace some of the Sr^2+^ ions due to charge compensation [156]. However, in a vast majority of RE^3+^ co-doped materials, different co-dopants yield distinct efficiency effects. For example, in Sr_2_MgSi_2_O_7_:Eu^2+^,RE^3+^ materials, co-doping with Dy^3+^ yields a 4-fold increase in the duration of the PeL, while Sm^3+^ co-doping decreases the PeL duration [158]. Dorenbos [147] proposed a mechanism for Sr_2_MgSi_2_O_7_:Eu^2+^,RE^3+^ materials suggesting that the energy level of the reduced form of the co-dopant (RE^2+^) act as an electron defect. In this proposed mechanism, the trapping of the electron by the co-dopant is responsible for reducing the co-dopant from the *3+* to the *2+* form. Thus, the divalent energy level positions related to the conduction band would determine the amount of thermal energy needed for depopulating the traps. Recently, Joos and co-workers [159] investigated the Sr_2_MgSi_2_O_7_:Eu^2+^, Dy^3+^ material and identified the reversible Dy^3+^ reduction during irradiation combining laser excitation and X-ray spectroscopy, proving that co-dopants act also as electron traps. This trapping property of the RE^3+^ co-dopants is efficient only when the energy level of the correspondent RE^2+^ ion is below the conduction band with appropriate energy. This phenomenon is absent in some materials like SrS:Eu^2+^,RE^3+^ [156] since the RE^2+^ ground states are either inside the conduction band or too deep compared to the bottom of CB (Figure 8).

Thus, efficient PeL materials design involves two parts, the presence of efficient activators and the high concentration of charge carrier traps with proper depth. The blue-green PeL emitting materials comprise the majority of the literature due to the low eye-sensitivity to longer wavelengths when adapted to dark [160], and the lack of efficient red emitters (with allowed transitions) that present efficient trapping [91]. Finally, there is the historical background, with most of the research being done using Eu^2+^. Eu^2+^ is a traditional blue-green emitter, where red emission requires doping in high crystalline field hosts or very covalent ones (due to the nephelauxetic effect). With a better understanding of the PeL mechanism and increased demand for applications in luminescence imaging of biological systems and solar energy harvesting, there is increased research on the design of new red and NIR-emitting PeL materials [91], Table 3. For extensive details on all PeL materials and different activators, the reader is advised other reviews [49,91,92,161]. In this review, we will focus solely on a few examples of the most common activators.

**Figure 9:**
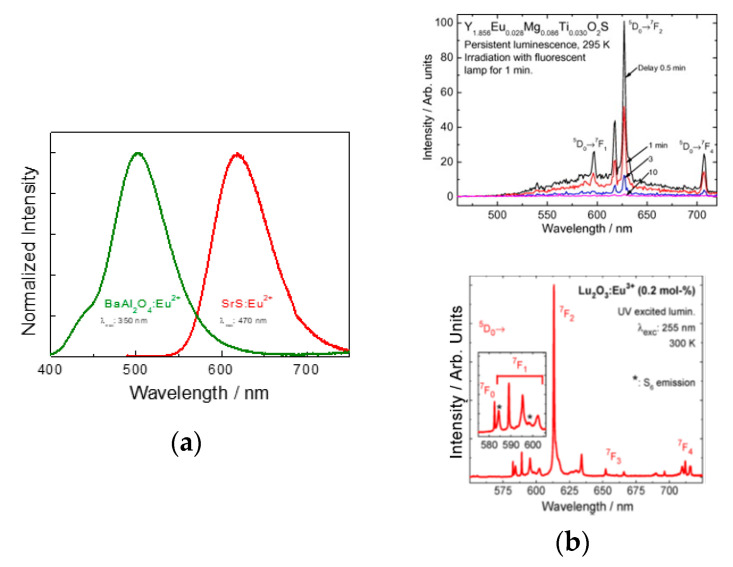
Emission spectra for (**a**) Eu^2+^ in two different hosts BaAl_2_O_4_ (left, green line) and SrS (right, red line) and (**b**) Eu^3+^ doped Y_2_O_2_S (top) [150] and Lu_2_O_3_ materials (bottom) [171]. Reproduced from [150] with permission from Elsevier. Reprinted with permission from [171]. Copyright (2016) American Chemical Society.

Even though there is a wide variety set of host-activator, only some elements from the *p*-, *d*- and *f*-block or crystalline defects are known to feature PeL in a crystalline host [161]. Among the *f*-block elements, the most efficient is Eu^2+^ [49]. PeL materials containing this ion exhibit emission ranging from the blue to the red regions of the electromagnetic spectrum (Figure 9a), depending on the crystal field and nephelauxetic effect. Its allowed Laporte 4*f^6^* 5*d^1^* → 4*f^7^* transition leads to high emission intensities. However, NIR emission is not possible in materials containing Eu^2+^ since it would require strong crystal fields or highly covalent environments [93]. NIR-emitting PeL materials are usually obtained using the 4*f*-4*f* transitions of trivalent lanthanides, for example, Pr^3+^, Nd^3+^, Er^3+^, and Yb^3+^. The only disadvantage of those materials is that the emission wavelength cannot be modulated due to the electronic shielding of the 4*f* orbitals by the 5*s* and 5*p* orbitals (Figure 9b) [142].

Cr^3+^ and Mn^4+/2+^ are the most explored *d*-block elements used as dopants on PeL materials [185,186,187]. PeL materials containing those ions feature emission due to *d*-*d* or charge transfer electronic transitions ranging from the visible to the NIR region of the electromagnetic spectrum. The *d*-*d* electronic transitions are forbidden by the Laporte rule and dependent on the crystal field. Thus, factors as coordination site symmetry, ligand field strength, and vibrionic coupling are essential for relaxing the Laporte rule, resulting in increased emission rates. For example, the *d*-*d* Cr^3+^-centered NIR emission in Cr^3+^-doped LaAlO_3_ perovskites and Cr^3+^-doped ZnGa_2_O_4_ spinels is due to the strong crystalline field on the hosts mentioned above [180,188]. Bi^3^+ and Pb^2+^ are the most explored *p*-block metals used as dopant in PeL materials due to their allowed metal-to-metal (MMCT), ligand-to-metal (LMCT), or 6*s*^2^ → 6*s*^1^ 6*p*^1^ electronic transitions [189].

### 3.2. Synthesis of PeL Nanomaterials

As aforementioned, a defined solid-state structure is a paramount factor in obtaining efficient and long-lasting PeL materials. Due to the need of controlling and optimizing two different outputs, the optical properties (i.e., high quality on excitation/emission spectra with a bright and long-lasting afterglow emission), and the particle size control (i.e., narrow size distribution and controlled morphology), synthesis of PeL materials are more challenging when compared to larger sized nanoparticles. Factors such as optimization of (co-)dopants percentage on host, annealing temperature range, heating exposure time, phase purity, amount of intrinsic defects are some of the ones to be considered [190,191,192,193,194].

Even though there is a range of possibilities for PeL-NPs synthesis, up to now, there is not a universal and flawless method for preparing PeL-NPs featuring intense light-emission, controlled size distribution, and morphology of the NPs. The solid-state synthesis (ceramic synthesis) is the most common method of obtaining a PeL material. The solids precursors are mixed and heated up to high temperatures [195,196]. Bulk PeL materials based on aluminates [141,191,197,198,199,200], silicates [201,202,203,204,205], and other compounds [47,49,206,207,208] have well-defined synthesis using this process. This method is well-known, and the annealing step is necessary to yield crystal phase purity and enhance the amount of defects in the structure. Alternatives synthesis, mostly wet-chemical methods, allows better control of particle size and morphology; however, the low temperatures and shorter reducing times yield materials with shorter PeL emission lifetime and/or a weak emission. Other preparation methods such as combustion synthesis, sol–gel, co-precipitation, and hydrothermal are also widely used for synthesizing PeL-NPs. Each of those methods has its particularities, and the ideal synthetic parameters, temperature, heating rate, pressure, and concentration, involve extensive bench time work and are dependent on each specific material.

#### 3.2.1. Combustion Synthesis

**Figure 10:**
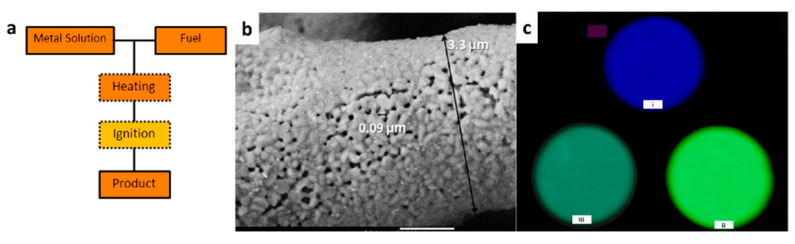
(**a**) Schematic flowchart of combustion synthesis (CS), and (**b**) SEM image of BaAl_2_O_4_:Eu^2+^, Dy^3+^ prepared using the CS method. Reproduced from [191] with permission from Elsevier. (**c**) Picture of the PeL emission of MAl_2_O_4_:Eu^2+^,RE^3+^ (M: (i) Ca^2+^, (ii) Sr^2+^ or (iii) Ba^2+^) prepared using the CS method. Reproduced from [215] with permission from Elsevier.

Combustion synthesis (CS), or self-propagating heating synthesis, is a low energy consuming method used to synthesize oxide ceramics that takes advantage of extremely exothermic reactions between metal nitrates and organic fuels (typically urea, carbohydrazide, or glycine) [209,210,211,212,213,214,215]. In a typical reaction, the synthesis occurs in a pre-heated muffle furnace, where the mixture of the nitrates and the organic fuel is inserted. As the synthesis initiates, the fuel ignites, rupturing into flames, and combustion takes place. The energy produced quickly heats the system (the temperature reaches values > 1000 °C) and sustains the temperature for a period of over 60 s, which is long enough to grow and crystallize the NP [209,210,211,212,213,214,215]. The final product is a fluffy, foamy powder with a large surface area (Figure 10a). The advantages of the CS method are its short reaction time, and the heating process tends to decrease undesired absorption of hydroxyl groups on the particle surface, which can act as a luminescent quencher depending on the PeL phenomenon. Another advantage is the extremely high temperatures achieved in short periods that reflect in increased concentration of defects, improving the energy storage capability of the material as shown by Rodrigues and co-workers for the blue-emitting material BaAl_2_O_4_:Eu^2+^,Dy^3+^ [191] (Figure 10b) and Qiu and co-workers for the MAl_2_O_4_:Eu^2+^,Dy^3+^ (M = Sr^2+^, Ba^2+^ or Ca^2+^) material [215] (Figure 10c). On the downside, the disadvantages of the CS method are the lack of reproducibility and difficulty in controlling the process due to the unpredictable combustion step resulting in a broad range of NP sizes.

#### 3.2.2. Sol–Gel Synthesis

**Figure 11:**
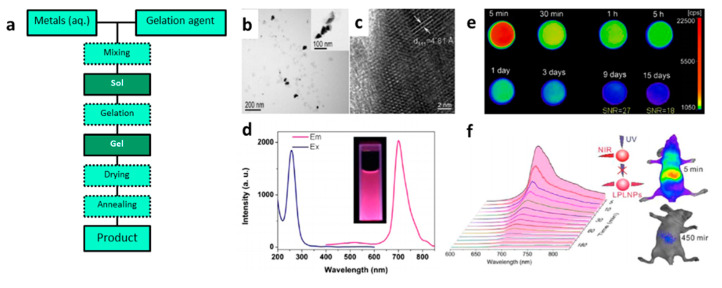
(**a**) Schematic flowchart of SGS. (**b**) TEM, (**c**) high-resolution TEM, (**d**) excitation (blue curve, left) emission at 700 nm) and emission (red curve, right) excitation at 254 nm) spectra of the aqueous dispersion of the material, (**e**) afterglow emission collected at different times after turning off UV excitation, and (**f**) in vivo NIR afterglow imaging. Material: Zn_2.94_Ga_1.96_Ge_2_O_10_:Cr^3+^,Pr^3+^, *λ_exc_* = 254 nm and *λ_em_* = 700 nm. Reprinted with permission from [219]. Copyright (2013) American Chemical Society.

Sol–gel synthesis (SGS) is a wet chemical technique widely used to prepare inorganic polymers and ceramics [216], including PeL materials. The sol–gel process is carried through a liquid solution, that eventually transforms into a sol, and finally into a viscous colloidal gel state. The main steps in SGS are the hydrolysis and/or condensation of molecular precursors (gelation agent), the formation of a sol–gel aqueous solution, suspension and drying of the solids, and annealing (Figure 11a) [216,217,218,219,220,221,222] Through the SGS technique is possible to produce a solid material from a homogenous solution. The SGS allows precise and flexible control when using precise synthesis conditions (reaction time, pH, temperature, the concentration of the precursors and surfactants, stirring, for example). Furthermore, SGS offers a precursor-homogeneity and a useful method for controlling the particle morphology and size. SGS is a widely used method for synthesizing aluminates and silicates based PeL-NP. For example, SrAl_2_O_4_:Eu^2+^,Dy^3+^ (SAO:ED) NPs by sol–gel synthesis using a mixture of the nitrate/acetate metals and citric acid as chelating agent [217]. The obtained SAO:ED NPs showed size in the 20 nm range, with a lasting afterglow centered at 520 nm. Sr_2_MgSi_2_O_7_:Eu^3+^,Dy^3+^ PeL-NP with an average size of 250 nm, were obtained using the SGS [220]. The advantage of the SGS for synthesizing this material is the use of tetraethyl orthosilicate (TEOS) as a silicon source. TEOS quickly goes through hydrolysis, which results in a viscous colloidal solution, reaching the required gel-state and being a physical limitation for particle growth. A similar route using citric acid was reported for synthesizing Zn_2.94_Ga_1.96_Ge_2_O_10_:Cr^3+^,Pr^3+^ NIR emitting PeL-NPs with persistent luminescence that lasted for over 360 h (Figure 11e), and size in the range 30–60 nm (Figure 11b,c) with good dispersibility in water (Figure 11d) allowing in vivo application (Figure 11f) [219].

#### 3.2.3. Co-Precipitation Synthesis

**Figure 12:**
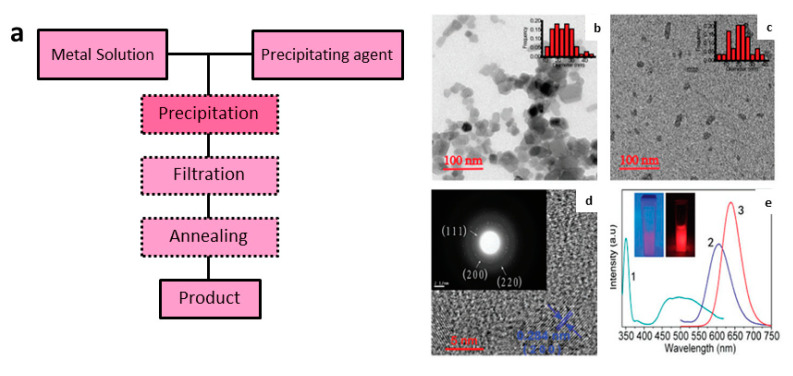
(**a**) Schematic flowchart of CPC. TEM images and (inset) histograms of the particle size distribution of (**b**) CaS:Eu^2+^, Sm^3+^, Mn^2+^ and (**c**) functionalized CaS:Eu^2+^, Sm^3+^, Mn^2+^. (**d**) HRTEM of CaS:Eu^2+^, Sm^3+^, Mn^2+^. The inset shows the SAED pattern. (**e**) Excitation (1 – green line, left), PeL emission (2 – purple line, right), and up-conversion emission (3 – red line, right) spectra. The inset shows photographs of CaS:Eu^2+^, Sm^3+^, Mn^2+^ under UV (left) and NIR excitation (right). *λ_em_* = 610 nm, *λ_exc_* = 355 nm (PeL) or *λ_exc_* = 980 nm (UC). Reproduced from [227] with permission from The Royal Society of Chemistry.

Co-precipitation synthesis (CPS) is based on the control of particle growth based on the solubility product constant of the precursors. This method relies on the solubility compatibility of starting materials, relatively low reaction temperature, and shorter synthesis time. This is a simple method where a saturated solution of soluble metals (most common are nitrates or acetates) is precipitated by the addition of a precipitant agent (e.g., urea, sodium silicate, sodium bicarbonate, for example) (Figure 12a). In general, the conditions that affect the CPS are the concentration of metals solution, the concentration of precipitating agent solution, the slow controlled mixture between both solutions, temperature when precipitating the solid and of the annealing process, and presence of complexing agents like EDTA (which affects the kinetics) [223,224,225,226,227,228]. Using the CPS method, Wang and co-workers synthesized water-dispersible nanocrystalline CaS:Eu^2+^,Sm^3+^,Mn^2+^ with 20–40 nm size range (Figure 12b–d), efficient PeL that also showed up-conversion properties (Figure 12e) [227].

#### 3.2.4. Hydrothermal Synthesis

**Figure 13:**
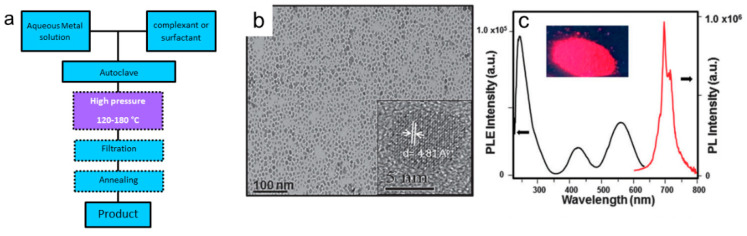
(**a**) Schematic flowchart of HS. (**b**) TEM image of ZnGa_2_O_4_:Cr^3+^ dispersed in hexane prepared via HS. (**c**) Excitation (black curve, left) and emission (red curve, right) spectra of the ZnGa_2_O_4_:Cr^3+^ dispersed in hexane. The inset shows the photograph of the PeL emission of the NP under 254 nm excitation (P = 6 W). *λ_em_* = 696 nm, *λ_exc_* = 254 nm. Reproduced from [230] with permission from The Royal Society of Chemistry.

Hydrothermal Synthesis (HS) refers to a wet chemical technique were the precursors are sealed and heated into reaction vessels (autoclaves). HS is carried out at high pressures, provided by the autoclave reactor, where the synthesis between precursors is promoted. A typical NP synthesis using the HS method occurs within a two-phase reaction medium, composed of two immiscible solutions, an aqueous solution containing the metal precursors and an organic solvent (e.g., toluene) containing a complexing or surfactant agent, like oleic acid, EDTA, or cetyltrimethylammonium bromide (CTAB) for achieving control over the nanocrystalline size and morphologies. As the system heats up and the pressure builds up, the solutions are perturbed, and the precipitation occurs at the liquid-liquid surface. After that, the system is cooled down, and the precipitant is centrifuged. The solid is then exposed to a high-temperature annealing treatment. This approach enables the synthesis of highly crystalline nanomaterials under relatively mild conditions (Figure 13a). Concentration, pH, annealing temperature, pressure, and reaction time are all factors that affect the HS [229,230,231]. For example, synthesis of ZnGa_2_O_4_:Cr^3+^ using the HS led to monodisperse PeL-NP with size in the sub-10 nm range (Figure 13b), and afterglow NIR emission (ca. 696 nm) (Figure 13c) longer than 40 min [230]. Some examples of PeL materials and NP size, synthesis method, emission wavelength, and afterglow duration are shown in Table 4.

In addition to the aforementioned methods, other methodologies like the template method [232,239,241], solvothermal method [228,234,237,240], electrospinning method [235,236], and laser ablation/deposition techniques [238] are capable of producing PeL-NP. Nevertheless, there is still a need for developing more controlled methodologies for preparing PeL.

## 4. Persistent Luminescence in Luminescence Imaging of Biological Systems

Due to its afterglow, PeL materials are desirable for luminescence imaging of biological systems due to the possibility of obtaining high-quality images with non-interference from the background [42,43,45,46,48,50,51,52,53,54,64,242,243]. When using PeL in luminescence imaging, two main approaches are taken into account, materials with ultra-long persistent luminescence irradiated (or charged) outside the organism or materials irradiated inside the organism that are reactivated with X-ray or NIR radiation. Finally, detecting the persistent luminescence out of the biological system requires emission in the red and NIR-emitting regions of the electromagnetic spectrum due to the low absorption by tissues and cells in this region [66]. In this review, we will present the recent literature on PeL used in cellular imaging, separating the materials as a function of the excitation source used to produce the PeL phenomenon.

### 4.1. Excitation in the UV

UV radiation is the most common excitation source for PeL nanomaterials since most lattice, and defects activators rely on high energy band gap and charge transfer transitions. Due to UV light’s low penetrability in tissues and cells, UV activated PeL materials have to be activated before incubation. Thus, exceptionally long afterglow is required from those materials, as the excitation is hampered after in vivo injection. To optimize UV-excited PeL materials application in luminescence imaging of biological systems, emission in the NIR is a must due to the low absorption of cells and tissues in this region that leads to improved signal-to-noise ratio. Gallates and germanates doped with Cr^3+^, a NIR activator, are frequently used in PeL imaging studies due to their optimal crystalline field [47] and defect structure [244].

**Figure 14:**
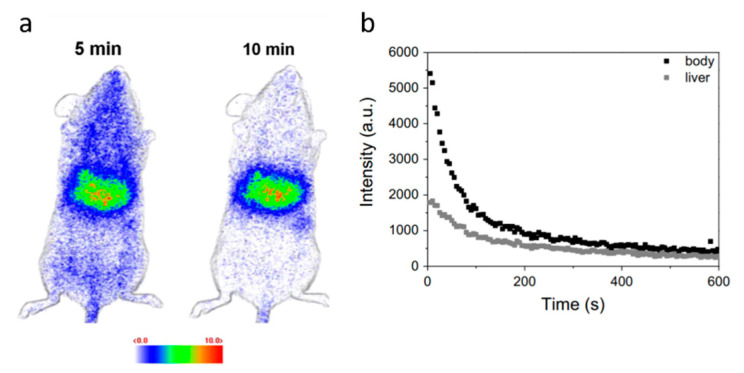
(**a**) In vivo imaging obtained at 5 and 10 min after the injection of the PeL-NPs. (**b**) Emission intensity as a function of the time monitoring the whole body and liver during the first 10 min of experiment. Pel-NPs: HAp/β-TCP doped Eu^2+^/Eu^3+^, Mn^2+^ and Dy^3+^. [PeL-NPs] = 0.8 mg/200 μL glucose. Reproduced from [245] with permission from Elsevier.

Maldiney and co-workers pioneered the use of NIR emitting PeL-NP in luminescence imaging of biological systems [48]. Using the PEG-functionalized ZnGa_2_O_4_:Cr^3+^ spinel PeL-NP the authors were able to obtain NIR-luminescence imaging of vascularization, tumors, and grafted cells, using UV excitation for 2 min at 254 nm before injection with decent accumulation in the tumor [48]. In follow-up work, the same research group improved the biocompatibility of the PeL-NPs by using hydroxyapatite/β-tricalcium phosphate (HAp/β-TCP) doped with Eu^2+^/Eu^3+^, Mn^2+^, and Dy^3+^, which exhibit efficient persistent luminescence for in vivo imaging after irradiation using UV excitation for 2 min at 254 nm (Figure 14) [245].

Using the same material, ZnGa_2_O_4_:Cr^3+^, Zhou and co-workers expanded the applications of PeL in luminescence imaging and demonstrated the application of biotinylated ZnGa_2_O_4_:Cr^3+^ PeL-NPs as a background-free luminescent nano-bio probe for sensitive and specific detection of avidin in a heterogeneous assay with a limit of detection of ~150 pM [240]. In the same year, Wang and co-workers demonstrated that functionalization of ZnGa_2_O_4_:Cr^3+^ NPs with hyaluronic acid (HA) and Gd_2_O_3_ yielded a multi-modal probe where high MRI contrast and high-quality NIR-PeL imaging were obtained for in vivo systems using UV excitation, at 254 nm before injection [246].

Besides the exciting PeL possibilities in luminescence imaging of biological systems, biocompatibility is still a challenge due to its low water solubility and low cell uptake. One of the most used strategies to remediate those limitations is surface functionalization with PEG, liposomes, or folic acid groups, which render improved water compatibility and cell uptake, respectively [247,248]. Another strategy is the functionalization with water-soluble polymers or dendrimers [249]. For example, Zhang and co-workers used the polyamideamine (PAMAM) dendrimer grafted on Zn_1.25_Ga_1.5_Ge_0.25_O_4_:0.5% Cr^3+^, 2.5% Yb^3+^, 0.25% Er^3+^ PeL-NPs surface for improved water solubility [249]. The dendrimer not only improves the water solubility but also allows multiple points for functionalization with other compounds. The PeL property was activated before the injection using UV light at 254 nm for 10 min, and the system was successfully used for in vivo imaging [249]. The use of the PAMAM allowed functionalization with Doxorubicin (DOX) via pH-sensitive hydrazine bonds resulting in the release under acidic conditions, characteristic of cancer cells but not healthy ones, resulting in decreased cell viability of HeLa cells and inhibition growth of tumors [249].

Although UV excitation of PeL-NPs before injection in biological systems has opened new avenues and demonstrated the potential of these materials for application in luminescence imaging of biological systems, it is not possible to activate these materials in vivo. That limits the applications to PeL materials that have a long afterglow.

### 4.2. Excitation in Visible

The success of UV-charged PeL-NPs in luminescence imaging of biological systems stimulated the development of PeL materials that could be activated in vivo or in vitro. Visible excitation in the far-red region of the electromagnetic spectrum has high penetrability due to the low scattering by cells and tissues. Thus, it is an alternative for expanding the use of PeL materials in luminescence imaging of biological systems.

**Figure 15:**
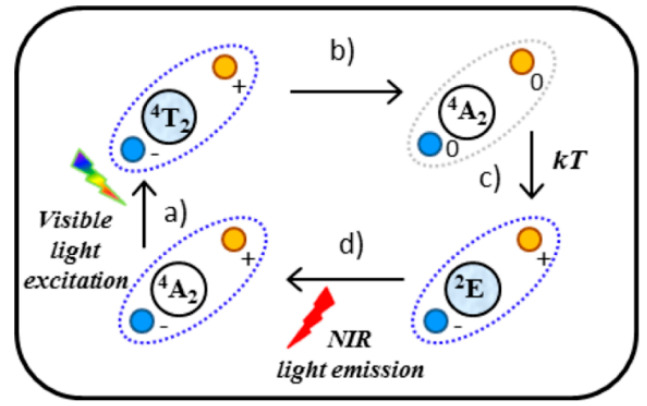
Proposed mechanism of PeL in ZGO:Cr induced by excitation below 3.1 eV. Cr_N2_ is represented by its states (^4^T_2_, ^4^A_2_ or ^2^E). Blue and yellow spheres represent the two opposite charge antisite defects. Steps: (**a**) optical excitation to the Cr^3+ 4^T_2_ excited level; (**b**) relaxation to the the Cr^3+ 4^A_2_ ground level, charge migration, and carriers trapping by neighboring antisite defects of opposite charges; (**c**) thermal release of e^-^-h^+^ pairs and trapping by Cr^3+^; (**d**) the Cr^3+ 2^E → ^4^A_2_ in the NIR. Reprinted with permission from [244]. Copyright (2013) American Chemical Society.

As described in Section 4.1 (vide supra), Maldiney and co-workers pioneered the use of NIR emitting PeL NPs in luminescence imaging of biological systems using the system ZnGa_2_O_4_:Cr^3+^ [48]. This material can also be activated using an orange-red LED source [48,206]. The mechanism that allows activation using an orange-red LED source was studied in detail by Bèssiere and co-workers and is related to antisite defects in the first neighborhood of a Cr^3+^ ion and differs from the usual PeL one (Figure 15) [244]. These defects are related to a swap between Zn^2+^ and Ga^3+^ sites in the crystal structure where Zn^2+^ substitutes a nearby Ga^3+^ in the spinel’s octahedral site, and Ga^3+^ replaces Zn^2+^ in the spinel’s tetrahedral site. This exchange causes a local charge imbalance where the octahedral and tetrahedral sites have negative and positive charges, respectively. The excitation of Cr^3+^ with visible light (^4^A_2_ (*t_2g_*)^3^ → ^4^T_2_ (*t_2g_*)^2^(*e_g_*)^1^ transition) leaves a hole and an electron in the *t_2g_* and *e_g_* orbitals, respectively forming an electron-hole pair. The nearby antisite defect pair drives the relaxation of Cr^3+^ back to the ^4^A_2_ ground state, storing the energy and rebalancing the charges of the defect. As a consequence, the tetrahedral and octahedral sites become neutral. This process is reversed through thermal energy, with Cr^3+^ going back to the ^4^T_2_ excited state and then relaxing to the ^2^E emitting state, responsible for the persistent emission in ca. 700 nm.

The possibility of using visible-light for charging PeL materials opened-up new avenues and expanded the number of PeL materials that could be used in luminescence imaging. For example, Shi and co-workers used the HS method and ethylenediamine as a solvent to obtain ZnGa_2_O_4_:Cr^3+^,Eu^3+^ PeL-NPs with -NH_2_ groups at the surface that were subsequently used to decorate the NP surface with either transacting activator of transduction peptide (TAT), or folic acid (FA). The first group, TAT-decorated, was successfully uptaken by HepG2 (liver cancer) and H22 (hepatocellular carcinoma) cells and was found to accumulate at the nuclei, while the FA-decorated NPs were successfully used to selectively target tumoral cells both in vitro (HepG2 cell line) and in vivo (H22 tumor-bearing mouse). Even in vivo, these PeL-NPs could be re-activated using a 650 nm or 808 nm LED, being excitation at 650 nm more effective [250]. In follow-up work, the same research group used 5 nm NPs with the same composition to target MCF7 cells [251]. FA-functionalization is a commonly used strategy for targeting cancer cells due to the overexpression of the folate receptor in cancerous cells. Li, Yan, and co-workers showed that FA-functionalization of Zn_1.25_Ga_1.5_Ge_0.25_O_4_: Cr^3+^, Yb^3+^, Er^3+^ PeL-NP were successfully used in luminescence imaging using a red LED source for in vivo excitation [252].

Long term toxicity is still an issue for in vivo applications of NP systems [248]. Sun and co-workers studied in detail the long-term toxicity of PEG-functionalized Zn_1.1_Ga_1.8_Sn_0.1_O_4_: Cr^3+^ PeL-NP. The advantage of using PeL in those studies is that it allows tracking in real-time using luminescence imaging without the constant need of a steady excitation source, allowing a detailed study of the pathway inside the body. The PeL-NPs were monitored for 60 days after injection, with regular tracking of the particles’ positions inside the body using the red excitation to recharge persistent luminescence. The NPs were found to accumulate in the reticuloendothelial system (RES), particularly lungs, liver spleen, and excretion through the digestive system. Histological, blood biochemistry and hematological analyses found no difference between the treated and non-treated mice [253].

Although the development of PeL-NPs with excitation in the visible was an improvement compared to UV-excited ones, the useful excitation wavelengths for in vivo applications are limited to the red and far-red wavelengths.

### 4.3. Excitation in the NIR

NIR excitation has attracted much attention due to its deeper penetration in the biological tissues [55,56,57,58,59,60,61,62,63,64,65,66,67,68,69,70,71,72]. Usually, the up-conversion (UC) phenomenon, followed by energy transfer, is used to induce persistent luminescence using NIR radiation [254]. In this case, it is challenging because it requires efficient UC emission and efficient energy transfer. Stimulated emission, using NIR excitation, is an alternative way to achieve PeL. In this process, NIR photons are used to bleach the populated traps (usually after UV irradiation).

**Figure 16:**
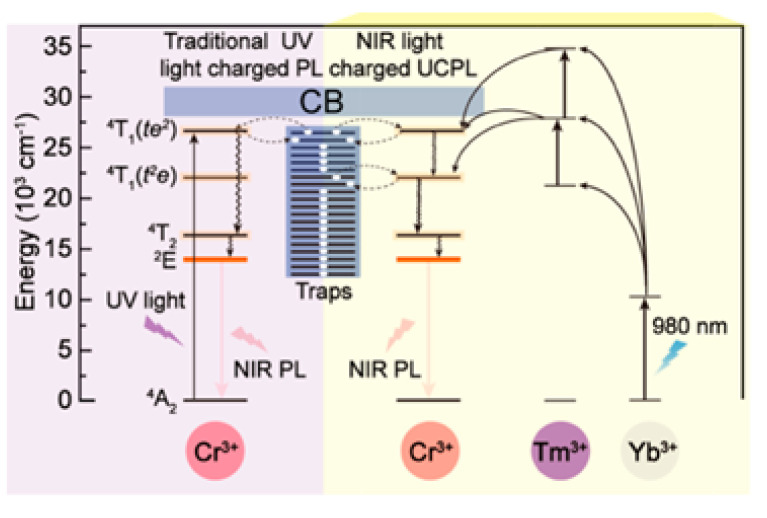
Energy diagram comparing the traditional UV charged PeL (left) and NIR-light-charged UCPL (right) mechanisms. Reprinted with permission from [257]. Copyright (2017) American Chemical Society.

The use of NIR light as an excitation source to induce PeL was first demonstrated by Liu and co-workers using Zn_3_Ga_2_GeO_8_ doped with Cr^3+^ and the UC pair Yb^3+^/Er^3+^ [255]. In this system, infrared excitation (980 nm) is used to populate excited states of Er^3+^. Through an internal energy transfer, the energy is transferred from Er^3+^ to Cr^3+^, and stored in defects in Cr^3+^ vicinities. Finally, with thermal energy aid, the Cr^3+^ excited levels are populated, and the energy is released over a long period through the Cr^3+^ characteristic emission. This phenomenon, named up-converted persistent luminescence (UPCL), was also used as a strategy in PeL luminescence imaging [256,257]. Xue and co-workers used the UPCL for demonstrating that PEG-functionalized Zn_3_Ga_2_GeO_8_:Cr,Yb,Er PeL-NPs could be readily recharged in vivo using excitation at 980 nm (150 mW × cm^−2^ for 120 s) with no efficiency loss after several cycles [256]. Conventional UC luminescence imaging was also possible using this system, allowing the development of synergistic probes taking advantage of both processes, UCPL and UC [256]. A multi-layered approach, composed of a self-assembled composite made of both PeL-NPs (Zn_1.1_Ge_1.8_Ge_0.1_O_4_:0.5% Cr^3+^) and UCNPs (*β*-NaYbF_4_:0.5%Tm^3+^@NaYF_4_) was proposed by Qiu and co-workers to ensure the efficiency of the UC, energy transfer, and PeL processes (Figure 16) [257]. Under excitation at 980 nm, the Tm^3+^ excited electronic levels are populated via an up-conversion energy transfer mechanism, followed by energy transfer to the PeL-NP, and finally, PeL at 700 nm. This hybrid material was used for tracking lymph nodes in mice [257].

Photostimulated emission is another way to obtain PeL using NIR excitation. In this process, the first step is the same as the conventional PeL phenomenon. The difference is that, instead of using thermal energy to bleach the traps, the system uses light energy to promote the charge carriers from the traps to the emitting center, generating the luminescence. For example, Gao and co-workers used the photostimulated luminescence of DSPE-PEG-biotin coated CaS:Eu^2+^,Sm^3+^ NPs for in vitro cellular luminescence imaging of HeLa cells. PeL is obtained using a white LED to excite the material, resulting in emission at ~650 nm. Excitation with NIR light is then used to produce photostimulated luminescence in this material after the original excitation, increasing the number of photons released while the light source is on [258].

### 4.4. Excitation in the X-ray

X-ray excitation has recently been proposed in the luminescence imaging of biological systems. Although there is still a small number of articles reporting X-ray induced PeL, these materials are promising for luminescence imaging [259,260,261,262]. The high penetrability of X-rays in cells and tissues allows, virtually, imaging of any part of the body, making this radiation attractive for in vivo applications. The high penetrability of the X-rays also allows recharging the PeL after hours, days, or even weeks after the PeL material injection avoiding the dependence on afterglow duration. The use of X-rays also opens up new avenues for combined luminescence imaging combined with X-ray absorption imaging [263].

**Figure 17:**
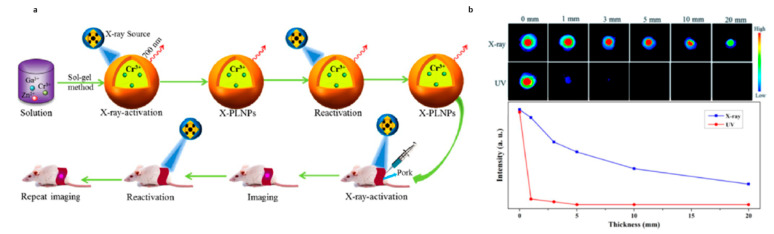
(**a**) Schematic diagram of in vivo PeL X-ray rechargeable luminescence imaging. (**b**) Phantom imaging as a function of time or pork tissue thickness (0, 1, 3, 5, 10, and 20 mm) using the PeL-NP ZnGa_2_O_4_:Cr^3+^. X-ray in vivo excitation for 5 min, at 45 kVp, or UV excitation prior to incubation for 20 min, at 365 nm. Reprinted with permission from [262]. Copyright (2017) American Chemical Society.

Xue and co-workers demonstrated X-rays’ high penetrability using the ZnGa_2_O_4_:Cr^3+^ PeL-NPs and comparing the luminescence imaging using UV for charging the NPs before injection or in vivo activation of the PeL using X-rays (Figure 17a) [262]. The use of X-rays not only allowed luminescence imaging of deeper tissues, when compared to UV (Figure 17b), but also allows recharging the PeL in vivo [262]. Strategies used to improve X-ray activated PeL materials usually involve doping or co-doping with heavy atoms such as Tb^3+^ and Sm^3+^ [263,264]. Zheng and co-workers recently demonstrated that X-ray activated MgGeO_3_:Mn^2+^,Yb^3+^,Li^+^ PeL-NPs have long afterglow and can emit in the first and second biological windows for long-term luminescence imaging [265].

### 4.5. Photodynamic Therapy Using Persistent Luminescence

**Figure 18:**
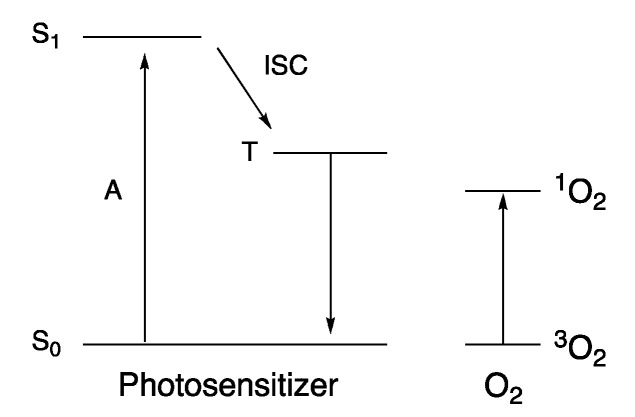
Energy level diagram illustrating the formation of ^1^O_2_. A denotes absorption, ISC intersystem crossing, S states with singlet and T states with triplet multiplicity.

PDT is a non-invasive therapy based on the generation of ^1^O_2_ and reactive oxygen species (ROS). The latter, generated through the interaction of the triplet level of a dye with ground state oxygen (^3^O_2_) (Figure 18), is used to damage cancerous cells [79,87,266,267,268,269,270,271,272,273,274]. Cells and organisms are less likely to develop resistance to ^1^O_2_, and it can therefore, be used successfully to treat cancer [79]. Organic dyes such as porphyrins, chlorins, phthalocyanines, and xanthenes are often used in PDT [87,88]. However, this class of compounds is prone to photobleaching, have low light-dark cytotoxicity ratios, and is also known to form aggregates that decrease the singlet oxygen generation efficiency as a function of the elapsed time, and thus decreases the efficiency of the treatment [89]. Additionally, the need for continuous in situ illumination causes damage to the skin and tissues.

**Figure 19:**
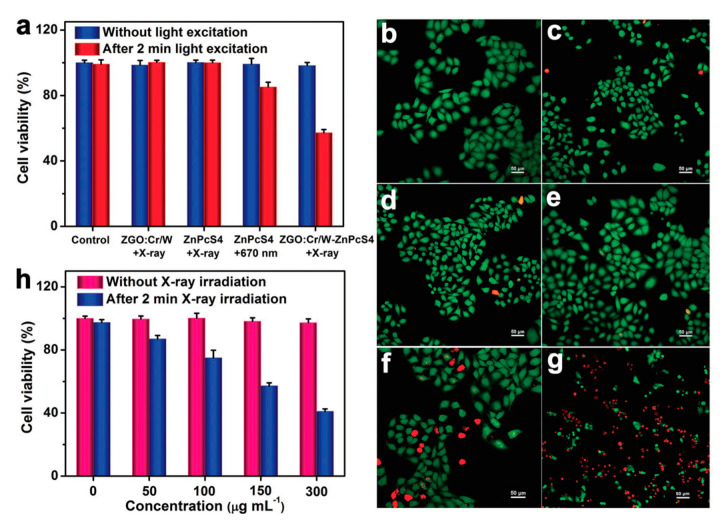
(**a**) HeLa cell viability without light excitation (blue bar) and after 2 min of irradiation (red bar). Luminescence imaging of HeLa cells treated with (**b**) PBS, (**c**) PBS + X-ray, (**d**) 150 μg mL^−1^ ZnGa_2_O_4_:0.5% Cr^III^, 0.5%W^VI^ + X-ray, (**e**) 5 μg mL^−1^ ZnPcS4 + X-ray, (**f**) 5 μg mL^−1^ ZnPcS4 + LED, and (**g**) 5 μg mL^−1^ ZnGa_2_O_4_:0.5% Cr^III^, 0.5%W^VI^–ZnPcS4 + X-ray. LED (*λ_exc_* = 670 nm, *P* = 160 mW cm^−2^). (**h**) HeLa cell viability without (pink bar) and after 2 min of X-ray irradiation (dark blue bar). The cells were treated with 150 μg mL^−1^ ZnGa_2_O_4_:0.5% Cr^III^, 0.5%W^VI^ + X-ray, 5 μg mL^−1^ ZnPcS4 + X-ray, 5 μg mL^−1^ ZnPcS4 + LED, and 5 μg mL^−1^ ZnGa_2_O_4_:0.5% Cr^III^, 0.5%W^VI^ – ZnPcS4 + X-ray. In the luminescence images, Calcein AM (green fluorescence) and propidium iodide (red fluorescence) indicates the living and dead cells, respectively. Reproduced from [282] with permission from Wiley.

The characteristic afterglow emission of PeL-NPs can be used as an internal light source in PDT that would eliminate the need for continuous in situ illumination, avoiding skin and tissue damage, and allowing the use of PDT in deep tissues. Curiously, the use of PeL in PDT is recent, and the first examples were reported back in 2016 [275,276]. In those pioneer works, the proof-of-concept that PeL could potentially be used in PDT was reported using ZnGa_2_O_4_:1% Cr^III^, 2% Pr^III^ as the PeL-NP, and the chemically bonded photosensitizer (PS) distyryl-BODIPY [275]. As noted by Akkaya and co-workers, only a modest photocytotoxicity against HepG2 cells was observed due to the short PeL emission lifetime in biological media. Re-charging the PeL is a strategy to repopulate the excited states of the PeL-NP and restore the PeL [276,277,278,279,280,281]. Solubilizing in water and targeting the PeL-NPs into cancer cells adds another challenge for in vivo PDT. Yan and co-workers proposed to study the effect of a cancer cell membrane (CCM) shell in the tumor accumulation using the system Zn_1.25_Ga_1.5_Ge_0.25_O_4_:0.5% Cr^III^, 2.5% Yb^III^, 0.25% Er^III^ as PeL-NP protected by a hollow SiO_2_ layer and loaded with DOX [280]. The CCM inhibits premature leakage and also yields targeting capability for metastases. As expected, the CCM shell’s presence yielded higher internalization than the system without it [280]. Due to the high absorption of cells and tissues, the wavelength used to re-charge the PeL-NP is within the biological window. Scherman, Richard, and co-workers reported that the PeL of ZnGa_2_O_4_:Cr^III^ can be restored using 808 nm excitation due to the UC excitation of the Cr^III^ [48]. Yan and co-workers incubated the system ZnGa_2_O_4_:Cr^III^ – Si-Pc in HepG2 cells for 8 h, and re-charged the PeL using 808 nm excitation pumps for 0, 3, 5, or 10 min that resulted in cell viability of almost 0 % (concentration = 200 μg × mL^−1^) proving the potentialities of using PeL-NPs in efficient PDT [276]. Although NIR radiation has a deeper penetration than to UV or visible wavelengths [61,63], it still cannot penetrate deeper tissues. X-ray radiation has unlimited penetrability, making this kind of radiation attractive deep tissue treatment using X-ray activated PDT (XPDT) [260,261]. Low dose X-ray radiation has been successfully used in PeL XPDT [282,283]. Yang, Li, and co-workers reported the photocytotoxic activity of ZnGa_2_O_4_:0.5% Cr^III^, 0.5%W^VI^ – ZnPcS4 in vitro against HeLa cells (Figure 19) and in vivo [282]. In this case, doping with W^VI^ enhances the X-ray cross-section absorption, and continuous ^1^O_2_ generation is observed over at least 40 min using X-ray radiation (0.09 Gy × min^−1^) [282]. The use of X-ray radiation increased the cytotoxicity compared to excitation at 670 nm (Figure 19a).

**Figure 20:**
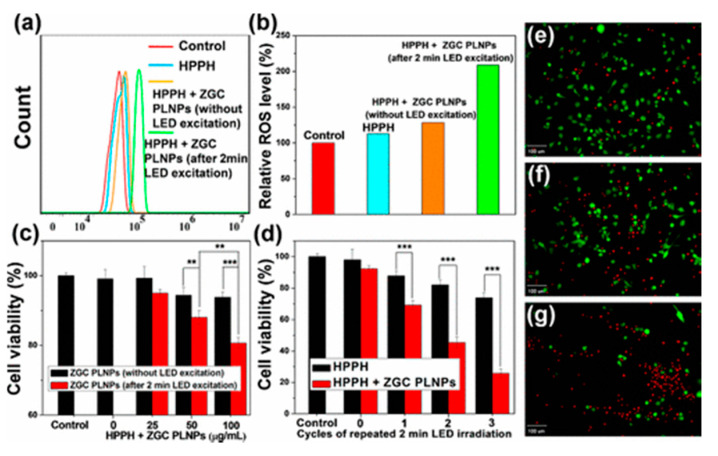
(**a**) Flow cytometry for intracellular ROS generation in U87MG cells. (**b**) ROS level. (**c**) U87MG cell viability after treatment HPPH and HPPH + different concentrations of with ZnGa_2_O_4_:0.4% Cr^III^ without (black bars) or with (red bars) light excitation. (**d**) U87MG cell viability after treatment HPPH and ZnGa_2_O_4_:0.4% Cr^III^–HPPH after several cycles of irradiation. Luminescence imaging of U87MG cells treated with 1 μg mL^−1^ HPPH + 50 μg mL^−1^ ZnGa_2_O_4_:0.4% Cr^III^ after (**e**) one, (**f**) two or (**g**) three cycles of 2 min irradiation. In the luminescence images, Calcein AM (green fluorescence) and propidium iodide (red fluorescence) indicates the living and dead cells, respectively. Reprinted with permission from [279]. Copyright (2017) American Chemical Society.

As highlighted above, long-lasting PeL is one of the most critical requirements for using PeL-NPs in PDT. One of the challenges is to develop less chemically aggressive synthetic routes that damage the PeL-NPs surface, causing a decrease in the PeL emission lifetime. An additional challenge for application in biological systems is the extensive emission quenching caused by the solvent. Synthetic methodologies to achieve hydrogels, hollow silica interlayers or hollow cavities with controllable size aim to achieve long-lasting PeL and improve cell biocompatibility [278,279,280,281]. For example, tumor-injectable oleosol implants are obtained by dissolving the PeL-NPs in a mixture of poly(lactic-*co*-glycolic acid)/*N*-methylpyrrolidone [279]. The injected oleosol quickly turns into a solid upon injection, and due to the decreased surface defects, long-lasting PeL is achieved [279]. In vitro and in vivo photocytotoxic activity against U87MG cells was demonstrated using the oleosol system containing ZnGa_2_O_4_:0.4% Cr^III^–HPPH showed (Figure 20) [279]. Although the use of oleosol injectable PeL-NPs systems leads to improved PeL, the solidification of the PeL-NP in the tumor and the fact that the PS is not chemically bonded to the PLNP may lead to undesirable accumulation in the body and leakage, respectively. The use of hollow structures seems to be a better approach for improving the PeL emission lifetime. In this approach, a ZnGa_2_O_4_:1% Cr^III^ shell is grown on the surface of carbon spheres. During the calcination process, the carbon core is burned, yielding hollow cavities. Loading of the cavities with DOX and Si-Pc and coating with BSA allow the use of this system for combined chemotherapy and PDT [281]. In solid tumors, the low concentration of O_2_ poses an additional challenge for PeL PDT. Some strategies reported to overcome the low concentration of O_2_ are the use of CaO_2_ in the structure of the system [277], generation of ROS by hydroxyl groups on the surface, or doping with Fe^III^ [277,284].

**Figure 21:**
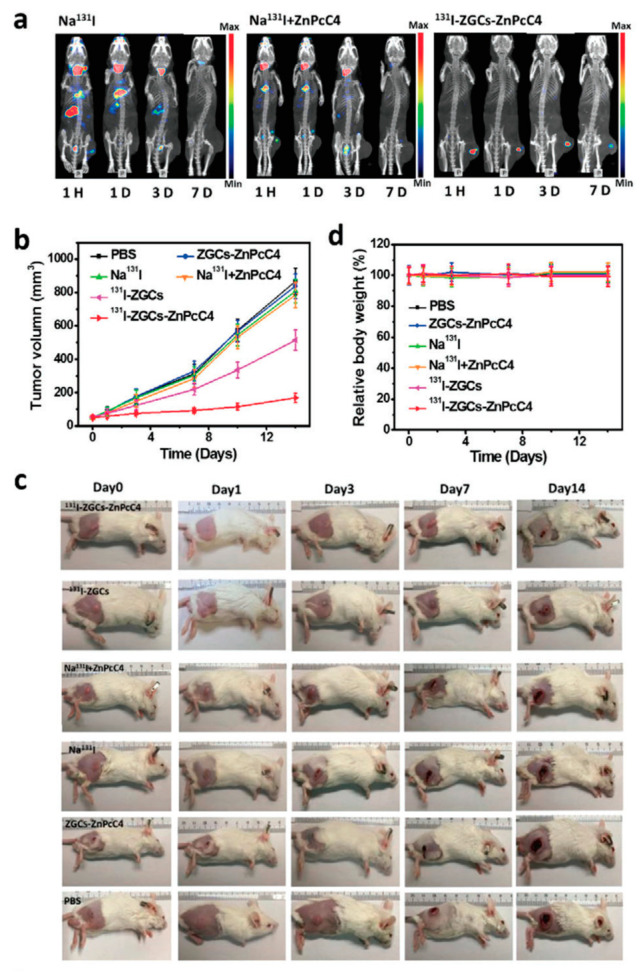
(**a**) SPECT/CT images of 4T1 tumor bearing mice treated with intratumoral injection of 100 μCi Na^131^I, 100 μCi Na^131^I + 20 100 μg ZnPcC4, and ^131^I-ZnGa_2_O_4_:Cr^III^–ZnPcC4 (100 μCi, 200 μg). (**b**) Tumor growth curves as a function of time for different treatments. (**c**) Representative photographs for different ice with different treatments. (**d**) Body weight as a function of time for different treatments. Reproduced from [289] with permission from Wiley.

All the examples discussed above are exciting and point to a bright future for PeL PDT. However, the need for “re-charge” the system is not ideal and deviates from the dream of having PDT without any external stimulation other than the initial charge. One of the radionuclide decay products is high-speed charged particles that move faster than the light in that medium, originating a faint luminescence in the UV-blue region of the electromagnetic spectrum called Cerenkov luminescence. Thus, the development of systems containing radionuclides yields an internal light excitation source [285,286,287,288]. Sun, Su, and co-workers recently reported using Cerenkov luminescence to generate PeL using the system ^131^I–ZnGa_2_O_4_:Cr^III^–ZnPcC4 [289]. Upon decay of ^131^I, a radionuclide used in radiotherapy, Cerenkov luminescence is generated and absorbed by the PLNP that produces PeL and excite the PS generating ^1^O_2_. Thus, yielding a system capable of treating diseases using combined radiotherapy and PDT [289]. No leakage of ^131^I and ZnPcC4 was observed in aqueous solution for over 7 days, which confirmed the stability of the ^131^I–ZnGa_2_O_4_:Cr^III^–ZnPcC4 system [289]. Extensive photocytotoxicity in vivo and in vitro against 4T1 cells was observed, in the absence of external light stimulation, for the ZnGa_2_O_4_:Cr^III^–ZnPcC4 system when compared with ZnPcC4, Na^131^I, or Na^131^I+ZnPcC4 (Figure 21).

## 5. Closing Remarks and Perspectives

The recent literature on PeL materials shows the wide variety of possible applications in the fields of luminescence imaging and photodynamic therapy to aid in the understanding of biological processes, diagnose, or treat diseases. The critical property of long emission for hours after ceased excitation allow these materials to shine in vivo, allowing better detection due to high noise/background noise ratio. This property could also be thought for substituting some radioactive markers diagnosis, leading to safer and cheaper exams. Although PeL eliminates the background interference, a challenge remains regarding the need for the emitted light to escape the biological systems. In an effort to solve this problem, there is now a high demand for the development of PeL materials that can be charged and emit in the NIR due to the high penetrability and low scattering of this light. To accomplish this goal, it is still necessary to combine the different aspects presented in this review: morphology control, long luminescence time, biocompatibility, and easy targeting.

The field of PeL-PDT is expected to have fast development in the coming years. The possibility of achieving a treatment that requires light, namely PDT, without the need for continuous excitation, is exciting and will advance non-invasive therapies. Achieving this goal will take first, the development of PeL-PDT systems with optimized ^1^O_2_ efficiency, second, the use of light with higher penetrability to allow deep tissue and in vivo treatment, and third, the development of Pel_NPs with specific targeting abilities to yield high accumulation in the cancer cells. To the date, only a few examples of PeL-PDT systems are known.

## Figures and Tables

**Table 1:** Commonly used dyes for fluorescence cell staining, organelle where the dye accumulates, and excitation and emission wavelength peaks [108].

Dye	Staining of	*λ_exc_*/nm	*λ_em_*/nm
Hoechst 33342	Nucleus	346	460
DAPI	Nucleus	359	461
NBD C_6_-ceramide	Golgi	466	536
DiO perchlorate	Cell membrane and lipids	488	510
BODIPY FL	Lipids	503	512
Rhodamine 123	Mitochondria	488	515
MitoTracker™ Green FM	Mitochondria	490	516
LysoTracker™ Red DND-99	Lysosomes	577	590

*λ_exc_* and *λ_em_* are the excitation and emission wavelengths, respectively.

**Table 2:** Excitation (*λ_exc_*) and emission (*λ_em_*) wavelengths peaks, and emission lifetimes (*τ*) for some of the cell components and dyes used in cellular luminescence imaging [133,134,135,136,137,138,139,140].

Compound	*λ_exc_*/nm	*λ_em_*/nm	*τ*/ns	References
NAD(P)H free	340	470	0.3	[133]
Flavin mononucleotide	444	558	4.27–4.67	[134,135]
Collagen	280–350	370–440	≤5.3	[133,136]
Riboflavin	420–500	520–750	4.12	[134]
Phenylalanine	258	280	7.5	[137]
Tyrosine	275	300	2.5	[138]
DAPI ^[a]^	359	461	2.78	[139]
Rhodamine 123 ^[a]^	488	515	3.97	[140]

[a]—in water.

**Table 3:** Examples of PeL materials containing different activators and their emission wavelengths [47,49,150,151,153,154,162,163,164,165,166,167,168,169,170,171,172,173,174,175,176,177,178,179,180,181,182,183,184].

Activator	Emission Wavelength	References
Defects	UV–NIR	[154,162,163,164,165,166]
Eu^2+^	Blue–red	[49,167,168]
Dy^3+^	Blue–red	[169]
Gd^3+^	UV	[170]
Eu^3+^	Red	[150,167,171]
Tb^3+^	Green	[171,172,173]
Sm^3+^	Red	[154,174]
Er^3+^	Red–NIR	[175,176]
Pr^3+^	Red–NIR	[154,177,178]
Yb^3+^	NIR	[151,179]
Cr^3+^	NIR	[47,180]
Mn^2+^	Green, yellow or red	[181]
Mn^4+^	NIR	[153]
Bi^3+^	Blue or NIR	[182,183]
Pb^2+^	UV	[184]

**Table 4:** Examples of PeL compounds, average size, synthesis method, emission wavelength (*λ_exc_*), and afterglow duration.

Compound	Average Size/nm	Synthesis Method	*λ_em_*/nm	Afterglow	Reference
CaAl_2_O_4_: Eu^2+^, Nd^3+^	70–80	co-precipitation	436	>360 s	[228]
	50	template	445	>2000 s	[232]
CaAl_2_O_4_: Eu^2+^, La^3+^	44	combustion	440	>800 s	[213]
Sr_2_MgSi_2_O_7_:Eu^2+^,Dy^3+^	20	combustion	457	>1800 s	[233]
	270	sol–gel	480	>1800 s	[220]
BaAl_2_O_4_: Eu^2+^,Dy^3+^	85–94	combustion	505	>20,000 s	[220]
CaS:Ce^3+^	42	co-precipitation	507	>200 ms	[225]
SrAl_2_O_4_:Eu^2+^,Dy^3+^,Tb^3+^	50–80	combustion	513	>2700 s	[213]
SrAl_2_O_4_: Eu^2+^,Dy^3+^	30	combustion	516	>1800 s	[215]
	20	sol–gel	520	>200 s	[217]
	50	co-precipitation	513	>2.5 h	[224]
	300	solvothermal	512	>100 s	[234]
	300	electrospinning	509	>200 s	[235]
Zn_2_SiO_4_:Mn^2+^	200	sol–gel	520	>20 ms	[221]
BiPO_4_:Tb^3+^	80–200	electrospinning	545	>15 ms	[236]
BiPO_4_:Ce^3+^	80–200	electrospinning	545	>15 ms	[236]
CaMgSi_2_O_6_:Mn^2+^	60–70	sol–gel	585	>1200 s	[222]
SnO_2_:Eu^2+^	50–100	solvothermal	588	>1000 s	[237]
Ca_2_Si_5_N_8_:Eu^2+^,Tm^3+^	5	laser ablation	610	>2000 s	[238]
CaS:Eu^2+^,Sm^3+^,Mn^2+^	30	co-precipitation	613	>30 min	[227]
Y_2_O_2_S:Eu^3+^, Mg^2+^,Ti^4+^	80–150	hydrothermal	627	>1000 s	[229]
Y_2_O_2_S:Eu3+,Ca^2+^, Ti^4+^	80–150	hydrothermal	627	>1000 s	[229]
Y_2_O_2_S:Eu^3+^,Sr^2+^, Ti^4+^	80–150	hydrothermal	627	>1000 s	[229]
Y_2_O_2_S:Eu^3+^,Ba^2+^, Ti^4+^	80–150	hydrothermal	627	>1000 s	[229]
CaMgSi_2_O_6_:Eu^2^, Pr^3+^,Mn^2+^	100	template	660	>1 h	[239]
ZnGa_2_O_4_:Cr^3+^	8	hydrothermal solvothermal	696 695	>3000 s >120 min	[230] [240]
Zn_3_Ga_3_Ge_2_O_10_:Cr^3+^,Pr^3+^	30–60	Sol–gel	695	>360 h	[219]

The background color on the *λ_em_* column represents the emission color of the PeLNPs.
